# Clinical Improvement Following Stroke Promptly Reverses Post-stroke Cellular Immune Alterations

**DOI:** 10.3389/fneur.2019.00414

**Published:** 2019-05-01

**Authors:** Antje Vogelgesang, Carl Witt, Christin Heuer, Juliane Schulze, Juliane Gellrich, Bettina von Sarnowski, Sönke Langner, Alexander Dressel, Johanna Ruhnau

**Affiliations:** ^1^Department of Neurology, University Medicine, Greifswald, Germany; ^2^Department of Diagnostic Radiology and Neuroradiology, University Medicine, Greifswald, Germany; ^3^Department of Neurology, Carl-Thiem-Klinikum, Cottbus, Germany

**Keywords:** stroke, immunosuppression, tissue plasminogen activator, HMGB-1, miRNA

## Abstract

**Background and Purpose:** Stroke induces immediate profound alterations of the peripheral immune system rendering patients more susceptible to post-stroke infections. The precise mechanisms maintaining stroke-induced immune alterations (SIIA) remain unknown. High-Mobility-Group-Protein B1 (HMGB-1) is elevated for at least 7 days post-stroke and has been suggested to mediate SIIA. Patients with rapid clinical recovery of neurological deficits rarely develop severe infections. We therefore investigated whether rapid neurological recovery (either spontaneous or secondary to neurovascular recanalization therapy) alters the course of SIIA. National Institutes of Health Stroke Scale (NIHSS) served as surrogate marker for neurological improvement.

**Methods:** Fluorescence-activated cell sorting was used to define leukocyte subpopulations. C-reactive protein (CRP), procalcitonin (PCT), HMGB-1, GM-CSF; IFN-β, IFN-γ, IL-1β, IL-1RA, IL-2, IL-4, IL-5, IL-6, IL-10, IL-12, IL-17, IL-17F, IL-18, TNF-α, MIF, IL-8, MCP-1, MCP-4, MIP-3α, MIP-3β, Eotaxin, soluble IL-6 receptor, E-selectin, and P-selectin were analyzed by ELISA or Multiplex Assays. Serum miRNA expression changes were analyzed by qPCR.

**Results:** Cellular parameters were similar in the improved and non-improved cohort on admission. In patients with rapid clinical recovery absolute and relative leukocyte, neutrophil, and lymphocyte numbers normalized promptly overnight. In contrast, HMGB-1 serum levels did not differ between the two groups. Nine miRNA were found to be differentially expressed between improved and non-improved patients.

**Conclusions:** SIIA are detectable on admission of acute stroke patients. While it was assumed that post-stroke immunosuppression is rapidly reversed with improvement this is the first data set that shows that improvement actually is associated with a rapid reversal of SIIA demonstrating that SIIA require a constant signal to persist. The observation that HMGB-1 serum concentrations were similar in improved and non-improved cohorts argues against a role for this pro-inflammatory mediator in the maintenance of SIIA. Serum miRNA observed to be regulated in stroke in other publications was counter regulated with improvement in our cohort.

## Introduction

Post-stroke infections, predominantly pneumonia, are associated with increased mortality and impaired neurological outcome. In recent years, it has been clearly shown in both experimental stroke and stroke patients that these infections are closely related to stroke-induced immune alterations (SIIA) of the peripheral immune system (1, 2). SIIA develop within the first hours of ischemic stroke and persist at least for days up to weeks (3–5).

Stroke volume and stroke severity on admission as determined by NIHSS are associated with post-stroke infections (6) and patients with transitory ischemic attacks have significantly fewer infectious complications and show a milder immune response after stroke compared to patients with complete stroke (7) and it is likely that patients with initially severe ischemic stroke that improve after hyperacute treatment have a reduced risk of infections and will show milder immune alterations after stroke.

To date it is unknown how SIIA are maintained and how they are reversed. We have investigated whether SIIA are influenced by clinical improvement. We hypothesized that rapid clinical improvement is associated with a reversal of SIIA. If SIIA were self-maintained after the induction within the initial hours of stroke, the time course of cellular changes should be similar in patients with rapid neurological recovery compared to those with persistent neurological deficits. If SIIA required an ongoing signal from injured brain tissue to persist, it should be reversed rapidly in patients with neurological improvement. Therefore, we took advantage of the National Institutes of Health Stroke Scale (NIHSS) reduction from admission to day 1 as a marker for the neurological improvement. Experimental and human stroke studies delivered proof that the hypothalamic–pituitary–adrenal axis and the autonomic nervous system release catecholamines and glucocorticoids which results in stroke-induced immune suppression. These hormones and transmitters have a very short half-life and return to normal levels within 24 h after an initial increase induced by ischemic brain injury (6) while the functional deficits of the immune system persist (4).

In animal models High-Mobility-Group-Protein B1 (HMGB-1) has been suggested as an important mediator of SIIA (8, 9). Others and we have reported earlier that HMGB-1 is elevated post-stroke at least for 7 days while its decoy receptors sRAGE and esRAGE are not altered (5, 8). HMGB-1 binds DNA within the nucleus but can be passively released during brain cell death or actively secreted by immune cells as an alarmin. Post-stroke the amount of HMGB-1 correlates rather with the amount of leukocytes in the peripheral blood than with the brain lesion size (10). We quantified HMGB-1 in serum of the improved and non-improved stroke patient cohort on admission and on day 3 to determine its possible role for maintaining post-stroke immune suppression.

SIIA include quantitative and functional alterations of innate and adaptive immune cells. Granulocytes increase in number but lose their ability to fight bacterial infections through radical production and neutrophil extracellular trap release (3). Lymphocytes are lost from the peripheral blood of patients (4, 5). It is also known that IL-6 and CRP are significantly elevated in patients suffering from an ischemic stroke and that HLA-DR on monocytes is reduced (5, 11). We therefore quantified peripheral blood immune cells and a panel of cytokines, chemokines, and acute phase proteins within the serum of the improved and non-improved stroke patient cohort.

MiRNA are known to be important regulators of immune responses and several miRNA were found to be differentially expressed after stroke (12–14). We investigated fold changes of serum miRNA amounts on day 1 post-stroke between the improved and non-improved cohort.

## Materials

### Patients and Controls

A *post hoc* analysis was performed on patients included in a prospective explorative study. This study recruited 40 ischemic stroke patients from the dedicated stroke unit of the University Medicine Greifswald from 2015 to 2016. Blood was sampled on admission within 24 h of stroke onset as well as on days 1, 2, 3, 4, 5, and 7 thereafter.

Patients aged ≥18 years suffering from ischemic middle cerebral artery (MCA) stroke were eligible for this study within 12 h after the onset of symptoms if their National Institutes of Health Stroke Scale (NIHSS) was ≥8 and if no signs of systemic infection were detected on admission (C-reactive protein (CRP) ≤50 mg/L and procalcitonin (PCT) ≤0.5 ng/mL). Patients receiving immunosuppressive drugs or diagnosed with a non-curatively treated malignoma were not recruited. Additional exclusion criteria were severe cerebral disorder in medical history (e.g., ischemic or haemorrhagic stroke, meningitis, encephalitis, severe cerebral trauma, or epilepsy), any contraindication for MRI (e.g., pace maker), clinically significant anemia (hemoglobin < 3.7 mmol/l), immunosuppression and the lack of written informed consent by the patient himself or through a surrogate where appropriate (See supplemental Table I).

All patients received best medical care on a dedicated stroke unit according to local standards. Recanalization with recombinant tissue plasminogen activator (rtPA) and/or thrombectomy was carried out as clinically indicated. Age-matched control individuals were recruited from the ophthalmology clinics among patients scheduled to receive cataract surgery. Controls were neurologically and immunologically healthy. See the participants' characteristics in Table 1. The study was approved by the local ethics committee (No. BB 050/15).

**Table 1:** Patients characteristics.

**Variable**	**Patient group (*N* = 40)**	**Control group (*N* = 16)**	Improved^*^ (*N* = 13)	**Non-improved (*N* = 27)**
Age [Years, Mean ± Std.]	71 ± 14	71 ± 8	65 ± 16	73 ± 13
Sex [as % female]	55%	44%	69%	49%
BMI, Weight/Height^2^ [Mean ± Std.]	28.45 ± 5.13	27.30 ± 3.42	30.43 ± 5.87	27.48 ± 4.53
**CO-MORBIDITIES**				
Hypertension [*n* (%)]	31 (77.5)	8 (50)	8 (61.5)	23 (85.2)
Diabetes mellitus [*n* (%)]	6 (15)	5 (31.9)	0 (0)	6 (22.2)
**STROKE CHARACTERISTICA**				
**Etiology**				
Large-artery artherosclerosis	10 (25)	NA^#^	4 (30.8)	6 (22.2)
Cardioembolism [*n* (%)]	19 (47.5)	NA^#^	6 (46.2)	13 (48.1)
Stroke of other determined Etiology [*n* (%)]	2 (5)	NA^#^	0	2 (7.4)
Stroke of undetermined Etiology [*n* (%)]	9 (22.5)	NA^#^	3 (27.1)	6 (22.2)
1. MRI Stroke Size [ml^3^, Median (IQR)]	31.0 (11.9–78.8)	NA^#^	11.8 (4.5–23.8)^$^	61.5 (23.7–201.7)^$^
2. MRI Stroke Size [ml^3^, Median (IQR)]	40.5 (9.4–80.1)	NA^#^	8 (5.9–13.1)^$^	63.0 (19.2–89.8)^$^
Initial NIHSS [Median (IQR)]	15 (11.25–21.00)	NA^#^	15 (11–19)	15 (12–21)
Infarct side [*n* (%) left-sided infarcts]	20 (50.0)	NA^#^	6 (46.2)	14 (51.9)
Treatment [*n* (%)]	30 (75.0)	NA^#^	13 (100.0)^$^	17 (63.0)^$^
Systemic Thrombolysis [*n* (%)]^&^	25 (62.5)	NA^#^	10 (76.9)	15 (55.6)
Mechanical Thrombectomy [*n* (%)]^&^	18 (45.0)	NA^#^	11 (84.6)^$^	7 (25.9)^$^
Combined Treatment [*n* (%)]	13 (32.5)	NA^#^	8 (30.8)^$^	5 (18.5)^$^

& *The numbers of systemic thrombolysis and mechanical thrombectomies are the total number of patients receiving the treatments and include patients receiving a combination of both*.

NA,

# *Not applicable*.

$ *1. MRI Stroke: Mann Whitney test, p = 0.0170; 2. MRI Stroke: Mann Whitney test, p = 0.0120; Treatment: Fisher's exact test, p = 0.0164; Mechanical thrombectomy: Fisher's exact test, p = 0.007; Combined treatment: Fisher's exact test, p = 0.0114*.

* *Clinical improvement was defined as a reduction of more than 25% of the NIHSS score from admission to day 1*.

## Methods

### Blood Sampling

Blood was obtained immediately on admission (d0) and between 6:30 and 7:30 in the morning on days 1, 2, 3, 4, 5, and 7 in different quantities. Differential blood cell counts (XN9000, Sysmex, Norderstedt, Germany) and the values for PCT, IL-6 (Adivia Centaur XPT, Siemens Healthcare Diagnostics, Eschborn, Germany) and CRP (all measured with the Dimension Vista, Siemens Healthcare Diagnostics, Eschborn, Germany) were measured in the central laboratory facility of the University Medicine Greifswald.

### Determination of Cell Subset

Fluorescence-activated cell sorting was used to measure cell counts for leukocytes, neutrophils, T cells, CD4+T cells, CD8+T cells, NK cells, and B cells (anti-CD45 FITC; anti-CD56 PE, anti-CD16 PE, anti-CD19 ECD, anti-CD3 PC5, IgG1 PC7, anti-CD4 PE, anti-CD8 PCD, anti-CD3 PC5 [Beckman Coulter]).

### Determination of Inflammatory Mediators

All patients' sera were sampled on admission day (day 0), days 1, 3, and 5 and immediately frozen at −80°C for later analysis.

Biomarker determination was performed at the Multiplex Facility at University Leiden utilizing the multi-analyte profiling (xMAP) technology (Luminex, Austin, USA) for Cathepsin S, Eotaxin, E-selectin, GM-CSF; IFN-β, IFN-γ, IL-1β, IL-1RA, IL-2, IL-4, IL-5, IL-6, IL-8, IL-10, IL-12, IL-17, IL-17F, IL-18, MIF, MCP-1, MCP-4, MIP-3α, MIP-3β, P-selectin, MMP-3, soluble IL-6 receptor, TNF-α). To analyse stroke recovery effects and not inter-individual differences only those cytokines were included into the analysis where stroke patients with or without improvement had no differences on day of admission.

IL-6 was determined twice due to multiplex setup. We only show the results gained in the central laboratory facility of the University Medicine Greifswald which provide higher precision in terms of standard deviation and coefficient of variation.

HMGB-1 was determined by ELISA according to the manufacturer's instructions (IBL International, Hamburg, Germany) from serum samples gained and frozen at −80°C on admission and day 3. Haemolytic serum samples were excluded because they lead to false positive ELISA results. Some HMGB-1 levels exceeded the standard range and were set to the highest standard due to lack of sample for reanalysis.

### Determination of Stroke Lesion Size

Diffusion weighted MRI images were used to calculate infarct sizes (3.0 Tesla). To analyse the location and stroke size images were analyzed using OSIRIX 5.6. To calculate the infarct size, the regions of interest were defined manually and the lesion volume was calculated semi-automatically. Initial MRI was performed between days 1 and 3; the second MRI was performed between days 5 and 7 post-stroke.

### Statistical Analysis

Because this is a *post hoc* data analysis, group sizes were not calculated prior to analysis, but *post-hoc* power analysis suggests, that our group sizes are adequate to detect effect sizes from previous trials (4). All data sets were tested for adherence to the Gaussian distribution with the Kolmogorov-Smirnov test. Since some of the data failed the normality test we used non-parametric testing throughout. The Kruskal-Wallis test with Dunn's multiple comparison test as a post-test or the Mann-Whitney test were used as appropriate. For comparing dichotomous data fisher's exact test was used. Correlations were determined by Spearman analysis. GraphPad-PRISM 5.0 (GraphPad Software Inc., San Diego, CA, USA) was used for all analyses. A *p* < 0.05 was regarded as significant.

MiRNA Groups were tested for differential expression by applying a *t*-test. None of the 9 herein found regulated miRNA passed a Benjamini-Hochberg correction to avoid false positive results at a significance level of 0.05. The results of the *t*-test reported in this manuscript are therefore explorative.

### Definition of Clinical Improvement

**Figure 1:**
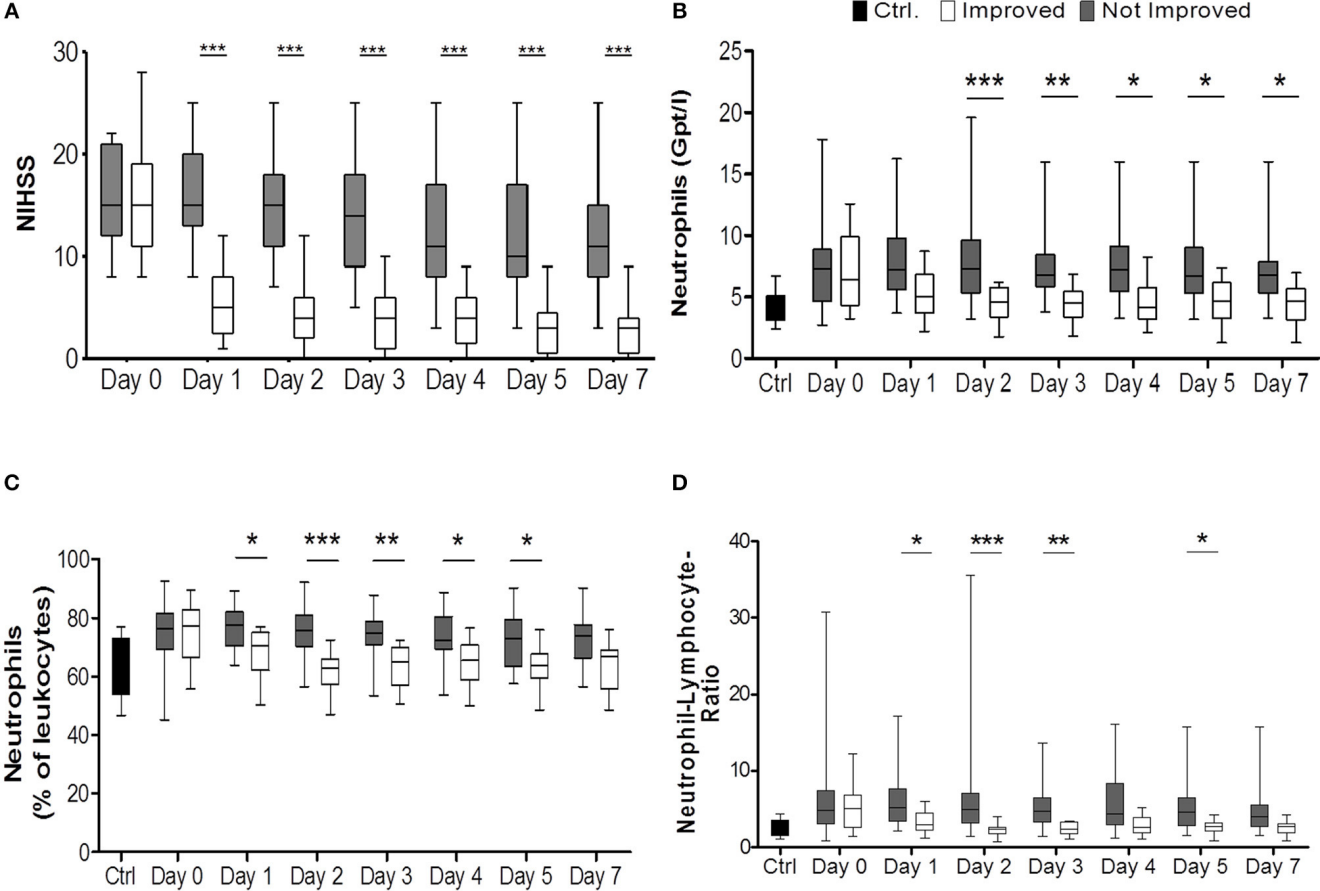
Comparison of improved patients (white bars) vs. non-improved patients (gray bars) on the day of stroke unit admission (day 0), days 1, 2, 3, 4, 5, 7. National Institutes of Health Stroke Scale (NIHSS) served as surrogate marker for neurological improvement **(A)** (n_imp_ = 13, n_non−imp_ = 27). Healthy control patients are given in black bars. **(B)** illustrates the absolute numbers of neutrophils (Gpt/l) (n_imp_ = 12, n_non−imp_ = 25) while **(C)** shows relative numbers (%) (n_imp_ = 12, n_non−imp_ = 25). In **(D)** the lymphocyte-neutrophil ratio is shown (n_imp_ = 12, n_non−imp_ = 25). *p < 0.05; **p < 0.01; ***p < 0.001; Box and whiskers (Min–Max) are given.

To study the effect of rapid clinical improvement on SIIA we took advantage of the NIHSS score and defined a reduction of more than 25% from admission to day 1 as marker of rapid clinical improvement after stroke. Thirteen patients did profoundly improve by this definition while 27 patients did not (see Table 1; Figure 1A).

### miRNA

We investigated fold changes of serum miRNA amounts on day 1 post-stroke between the improved and non-improved cohort. Serum miRNA was isolated from all patient samples and then analyzed by SYBR Green based miRCURY LNA PCR Human Panel I assay by Qiagen. The assay included 372 different miRNA. Samples were analyzed individually. Quality controls included melting curve analysis, calculation of amplification efficiency and comparison of Cq values to background level in the negative control sample. Spike-ins served as RNA isolation and cDNA synthesis control. To avoid data variation Quiagen controlled for hemolysis by the ratio between red blood cell miR-451 and miR-23a that is relatively stable in serum and plasma and not affected by hemolysis. Normalization was performed based on the average of the assays detected in all samples as this was shown to be the best normalization for qPCR studies involving numerous assays (15). For the present study, this included 43 assays.

## Results

Notably, all patients who improved rapidly after stroke according to our definition received some form of treatment, either mechanical thrombectomy or systemic thrombolysis with rtPA or both, while in the groups without rapid improvement only 63% of patients received some form of treatment. This difference was statistically significant (*p* = 0.0164). While both groups had nearly identical median NIHSS scores on admission [NIHSS_improved_: 15 (IQR 11–19), NIHSS_non−improved_: 15 (IQR 12-21)], median NIHSS scores were significantly different on all other days (*p* < 0.0001; Figure 1A). Stroke volumes measured by MRI after treatment and early improvement were markedly smaller for patients with clinical improvement compared to patients without clinical improvement (Table 1) between days 1 and 3 (*p* = 0.001) and days 5 and 7 (*p* = 0.012).

### Cellular Parameters

Comparison of the total stroke cohort with controls revealed changes in agreement with previous reports: Total leukocyte numbers increased significantly on all days compared to controls (Supplemental Figure IC), and absolute and relative neutrophilia (p_absolute_ = 0.0041, Figure 1C; *p*_relative_ = 0.0025, Supplemental Figures IA,B) was observed. Also, the absolute number of CD4+ T cells was significantly reduced on admission and on day 1 (*p* = 0.0345, Supplemental Figure IIA). CD8+ T cells, NK cells, and B cells weren't altered in absolute numbers or percentage of parent leukocytes (Supplemental Figures IIB–D).

On admission, the immune alterations observed in patients with subsequent improvement and without improvement were remarkably similar, no significant differences were observed for the cell populations investigated.

Improved patients had lower neutrophil counts and frequency than non-improved patients from days 1 or 2 until day 7 after stroke (*p*_absolute_ < 0.0001; *p*_relative_ < 0.0001; *post-hoc*: days 1, 2, 3, 4, 5) (Figures 1B,C, Supplemental Figures IIIB, IVA). The lymphocyte-neutrophil ratio was significantly increased in patients without improvement on days 1, 2, 3, and 5 (Figure 1D, Supplemental Figure IIID).

**Figure 2:**
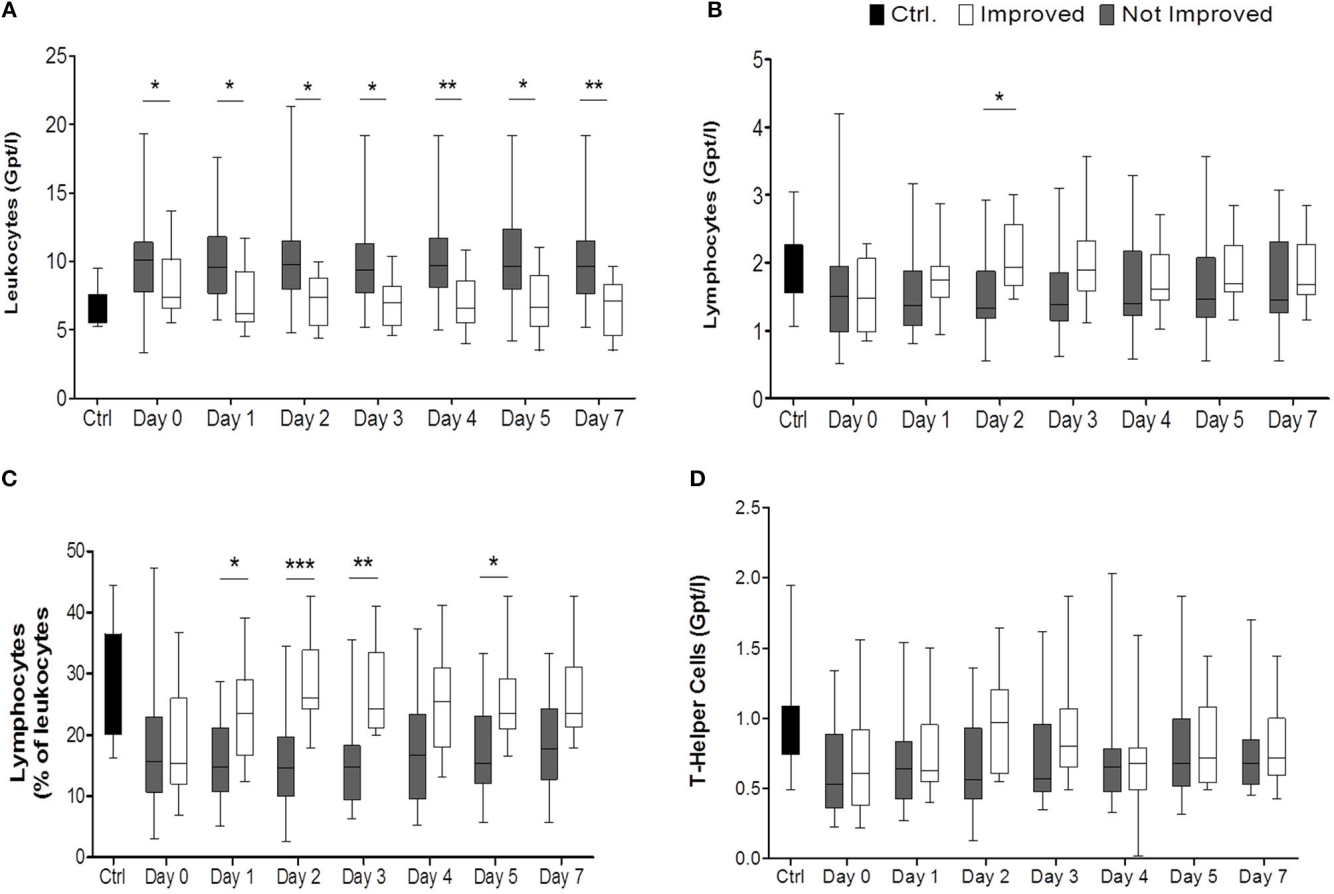
Comparison of improved patients (white bars) vs. non-improved patients (gray bars) on the day of stroke unit admission (day 0), days 1, 2, 3, 4, 5, 7, while healthy control patients are given in black bars. In **(A)** the absolute numbers of leukocytes (Gpt/l) (n_imp_ = 13, n_non−imp_ = 26), in **(B)** absolute numbers of lymphocytes (Gpt/l) and in **(C)** relative numbers of lymphocytes (%) are shown. Absolute T helper cells (Gpt/l) are given in **(D)**. *p < 0.05; **p < 0.01; ***p < 0.001; Box and whiskers (Min–Max) are given (n_imp_ = 12, n_non−imp_ = 25).

From days 1 to 7 absolute leukocyte counts were significantly elevated in the non-improved group compared to the improved one (*p* < 0.001) (Figure 2A, Supplemental Figure IIIA). Non-improved patients had more pronounced absolute and relative lymphocytopenia during the observation period than patients with neurological improvement (for absolute numbers: *p* < 0.0306, *post-hoc*: day 2; for relative numbers: < 0.0001, *post-hoc*: days 1, 2, 3, 5) (Figures 2B,C, Supplemental Figures IIIC, IVB). The absolute CD4+ T lymphocyte count tended to be higher in improved patients than in not improved patients especially on day 2 (Figure 2D). CD8+ T cells, NK cells, and B cell subpopulations did not differ between the two groups (data not shown).

### Acute Phase Proteins

**Figure 3:**
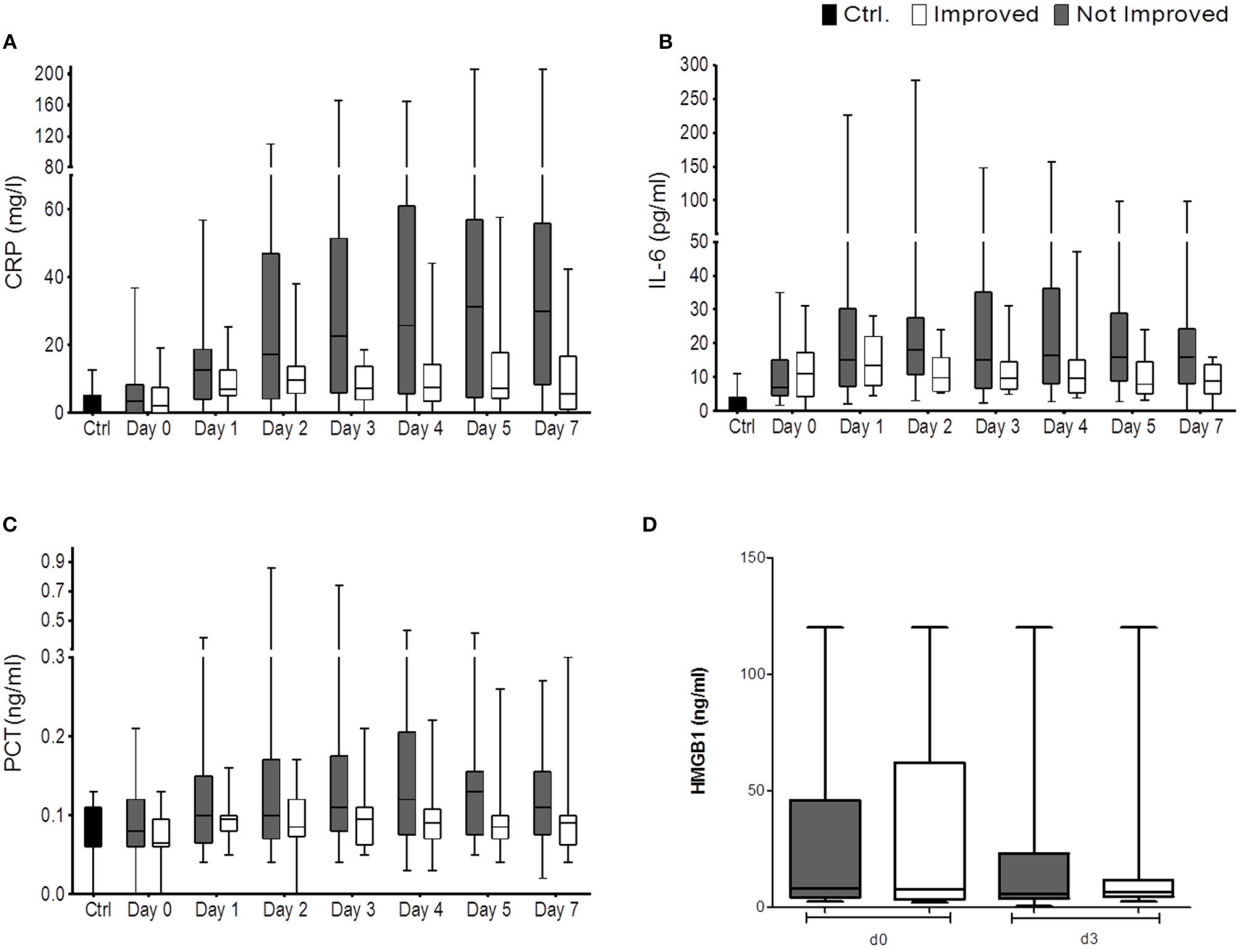
CRP (mg/L) **(A)**, IL6 (pg/ml) **(B)**, PCT (ng/ml) **(C)**, and HMGB-1 **(D)** are shown for the comparison of improved patients (white bars) vs. non-improved patients (gray bars) on the day of stroke unit admission (day 0), days 1, 2, 3, 4, 5, 7 (n_imp_ = 13, n_non−imp_ = 27). HMGB-1 (in ng/ml) is shown for improved patients (white bars) and non-improved patients (gray bars) on the day of stroke unit admission (day 0) and day 3. Box and whiskers (Min–Max) are given (n_imp_ = 11, n_non−imp_ = 16).

Despite an apparent trend toward higher CRP, PCT, and IL-6 in the non-improved cohort, statistical significance was not reached throughout the observation period (Figures 3A–C).

We found serum concentrations of HMGB-1 similar to our last study (5, 10). While the investigated immune alterations post-stroke had promptly reversed in the improved cohort, HMGB-1 concentrations were not differentially regulated between the improved and non-improved cohort at the investigated time points (Figure 3D).

### Serum Pro- and Anti-inflammatory Cytokines, Chemokines, and Biomarkers

To analyse the effect of clinical improvement on soluble immune markers we measured cytokines (GM-CSF; IFN-β, IFN-γ, IL-1β, IL-1RA, IL-2, IL-4, IL-5, IL-6, IL-10, IL-12, IL-17, IL-17F, IL-18, TNF-α, MIF), chemokines (IL-8, MCP-1, MCP-4, MIP-3α, MIP-3β, Eotaxin), and biomarkers (Cathepsin S, MMP-3, soluble IL-6 receptor, E-selectin, P-selectin).

**Figure 4:**
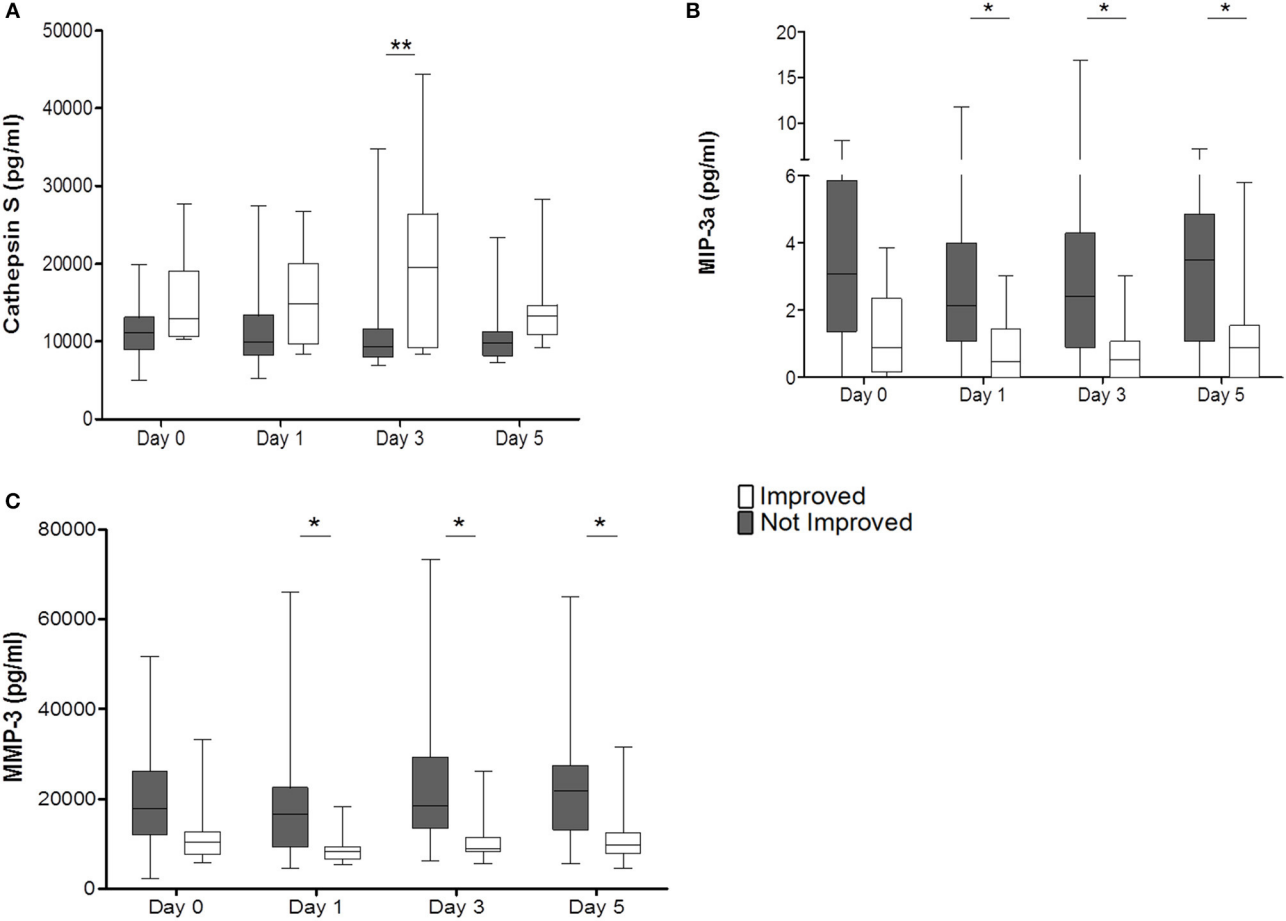
Improved patients (white bars) and non-improved patients (gray bars) are given for Cathepsin S (pg/ml) **(A)**, MIP-3a (pg/ml) **(B)**, and MMP-3 (pg/ml) **(C)**. *p < 0.05; **p < 0.01; Box and whiskers (Min–Max) are given (n_imp_ = 13, n_non−imp_ = 27).

Cathepsin S was significantly elevated in the improved cohort on day 3 (*p* = 0.0006) (Figure 4A). MIP-3a and MMP-3 were significantly reduced from days 1 to 5 in the improved cohort and also displayed the same trend on admission (MIP-3a *p* = 0.0001; MMP-3 *p* = 0.0003) (Figures 4B,C). Other cytokines, chemokines, and biomarkers were not regulated or showed inter-individual differences on admission (data not shown).

### Post-stroke Infections

We used a previously established definition of infections (4) to categorize patients. To fulfill these criteria patients had to show clinical signs of infection and have a serum CRP above 50 mg/L and PCT above 0.5 ng/mL. Only one patient in the non-improved cohort fulfilled all three criteria, suffering from pneumonia. Considering also patients fulfilling 1 or 2 criteria only (defined unclear infection status according to our criteria) 56% of the non-improved cohort had signs of infections, compared to 23% of the improved cohort.

### miRNA

Forty three of the Three hundred and seventy two analyzed miRNA were detectable in all samples investigates. The average number of miRNA detected per sample was 112. Nine miRNA were differentially expressed between the improved and non-improved patient group (cut-off *p* < 0.05) (Table 2).

**Table 2:** Top most differentially expressed miRNAs, showing the standard deviation (SD) across the groups, followed by average normalized Cq values for each group and fold change between the two groups.

**miR name**	**SD1**	**SD0**	**Average dCq 1**	**Average dCq 0**	**Fold change**	***p*-value**	**BH adj. *P*-value**
hsa-let-7e-5p	0.77	0.92	−3.7	−4.4	1.6	0.020	0.83
hsa-miR-431-5p	0.30	0.28	−4.9	−5.7	1.7	0.022	0.83
hsa-miR-584-5p	0.83	0.67	−4.1	−3.5	−1.5	0.030	0.83
hsa-miR-145-5p	0.67	0.83	−1.9	−1.3	−1.5	0.033	0.83
hsa-miR-30c-5p	0.51	0.51	−2.0	−2.4	1.3	0.034	0.83
hsa-miR-181a-3p	0.69	0.75	−5.7	−6.9	2.3	0.036	0.83
hsa-miR-99a-5p	0.64	0.92	−3.6	−4.2	1.5	0.037	0.83
hsa-miR-505-3p	0.86	0.93	−3.8	−4.5	1.7	0.040	0.83
hsa-miR-148b-3p	0.57	1.0	−5.2	−5.8	1.5	0.049	0.91

*The last two columns show the p-value from the t-test and the Benjamini-Hochberg adjusted p-value*.

## Discussion

Post-stroke immune alterations are a well-described phenomenon. Its functional characteristics have been investigated in different immune cell populations and its clinical relevance is revealed by the fact that in human and experimental studies the extent of SIIA correlates with post-stroke infection (4, 7, 16, 17). While stroke severity and the extent of SIIA correlate (10), the relationship of early neurological recovery and SIIA has not been investigated. Here, we describe the novel observation that early clinical improvement is associated with a rapid reversal of SIIA in stroke patients within 24 h. At admission, patients with and without improvement had identical stroke severity, as measured with the NIHSS score [15 (IQR 11–19) vs. 15 (12–21)]. As a rough estimate the NIHSS on admission can be considered a surrogate marker for the tissue at risk. Therefore, the neurological improvement can be considered to correlate with rescued penumbra. While the MRI imaging would allow for a better assessment of tissue at risk and the volume of brain tissue rescued due to recanalization therapy this MRI data on admission were not available for this study. Although we only measured stroke lesion size between days 1 and 3 (MRI 1) and days 5 and 7 (MRI 2) after stroke the observation that the MRI lesions of patients with an improvement are markedly smaller than in patients without improvement supports the use of these surrogate markers. Among patients with rapid improvement, the treatment protected viable brain parenchyma in the penumbra and thereby not only reduced the neurological deficit (NIHSS) but also reverted parts of the SIIA.

While the initiation of SIIA has been in the focus of many studies, data on the mechanisms that maintain or reverse SIIA remain scarce. HMGB-1 has been suggested to play a role in SIIA or influence the outcome of tested patients and animals in at least 6 studies (8–10, 18–20). With a given sample size that allows to detect large effect sizes only, we observed no difference in the regulation of HMGB-1 serum levels in patients with and without rapid clinical improvement. While our data do not support the hypothesis that HMGB-1 is involved in maintaining immunosuppression, additional studies are required to further define the role of HMGB-1 in stroke induced immunosuppression.

In our explorative analysis of miRNA expression we detected 9 miRNAs which were differentially regulated between improved and non-improved stroke patients. Five of these had been described as regulated in ischemic stroke compared to healthy controls. In agreement with our hypothesis, the miRNAs in the cohort of improved stroke patients appear to be regulated toward healthy controls. Of interest, miR99a which is enhanced in our improved cohort has been suggested to exert neuroprotective effects through inhibition of pro-caspase-3 and caspase-3 expression following cerebral ischemic stroke (21). Let-7e and miR-145 were reported to be upregulated by Sepramaniam et al. in acute stroke while miR-505 and miR-30c were reported to be downregulated (14). These miRNA were regulated in the opposite direction in our improved cohort accordingly.

MiRNA data regarding stroke are controversial discussed due to different methods, tissues, in man and animal studies (12–14, 21). Due to our small sample size our results are limited but proof the value of further investigations. Since miRNA seem to be differentially regulated between the improved and non-improved cohort they might emerge as possible targets for therapy to improve outcome especially in patients with an unsuccessful acute therapy.

Some limitations apply to our *post hoc* study. Due to the design of the original study the sample size is limited to 40 patients and no preceding sample size calculation was performed. As a consequence, resulting subgroups are small, limiting our power to the detection of large effects. Due to the exploratory nature of the study no adjustment for multiple testing was performed. Nevertheless, our findings are robust across the parameters investigated.

## Conclusion

While the course of stroke induced immunosuppression and the gradual recovery of immune function after the first week from stroke onset is well-described the change of the patients' immune status in dependence on successful acute recovery due to recanalization strategies had not been investigated. Here we report the novel finding that successful recanalization therapies by rtPA and thrombectomy not only improve neurological function but also reverse SIIA promptly. The rapid recovery observed in the improved cohort therefore strongly supports the requirement of continuous signals to maintain stroke induced immune alterations as early as day 1 post-stroke to maintain SIIA. Future studies should therefore focus on brain injury-induced signal cascades to fight the detrimental effects of SIIA in severe ischemic stroke.

## Ethics Statement

The study protocol was approved by the ethics committee of the Medical Faculty, University of Greifswald (No. BB 050/15). Controls gave direct informed and written consent to participate. Patients gave informed and written consent directly or through a surrogate.

## Author Contributions

AV, AD, and JR designed the study. CW, CH, and BvS recruited patients and controls. CW and CH evaluated the FACS and cytokine data. JG measured HMGB-1 and evaluated the data. SL evaluated stroke lesion size. JS, AV, AD, JR, and CW are responsible for data interpretation. AV, CW, JR, and AD wrote the manuscript.

### Conflict of Interest Statement

The authors declare that the research was conducted in the absence of any commercial or financial relationships that could be construed as a potential conflict of interest.
