# Corrigendum: Clinical Improvement Following Stroke Promptly Reverses Post-stroke Cellular Immune Alterations

**DOI:** 10.3389/fneur.2020.593366

**Published:** 2020-09-15

**Authors:** Antje Vogelgesang, Carl Witt, Christin Heuer, Juliane Schulze, Juliane Gellrich, Bettina von Sarnowski, Sönke Langner, Alexander Dressel, Johanna Ruhnau

**Affiliations:** ^1^Department of Neurology, University Medicine, Greifswald, Germany; ^2^Department of Diagnostic Radiology and Neuroradiology, University Medicine, Greifswald, Germany; ^3^Department of Neurology, Carl-Thiem-Klinikum, Cottbus, Germany

**Keywords:** stroke, immunosuppression, tissue plasminogen activator, HMGB-1, miRNA

A correction has been made to ***Acknowledgments*** in the original article to include Sonya Ceesay. The revised text appears as below:

**We thank the patients and their next of kin for participating in this study and stroke physicians for their support in the recruitment of patients. We thank Dr. Sabine Ameling for assistance in analyzing miRNA data. We thank Sonya Ceesay for supporting the study through control recruitment, blood sampling and data handling**.

The authors apologize for this error and state that this does not change the scientific conclusions of the article in any way. The original article has been updated.

