# Supplementary material for: Clinical Improvement Following Stroke Promptly Reverses Post-stroke Cellular Immune Alterations
Source: Front Neurol. 2019 May 1;10:414. doi: 10.3389/fneur.2019.00414 (PMC6504832; doi:10.3389/fneur.2019.00414)
Supplement: Supplementary file 1 [file Data_Sheet_1.pdf]

Supplemental Table I – Inclusion and exclusion of patients

50 patients/ 16 controls

<u>10 patients excluded:</u>

2 withdrawn consent

2 hemicraniectomy

1 abnormally high B cell count

2 no anterior circulation stroke on MRI

3 suspected infection on admission day

40 patients/ 16 controls
Analysed in this study

Inclusion criteria	Exclusion criteria
– Clinical signs of cerebral ischemia in middle cerebral artery (MCA)<br>– Stroke admission within 24 hours after onset of infarct signs<br>– NIHSS ≥ 8<br>– Age ≥ 18 years<br>– No immune suppressive drugs<br>– CRP ≤ 50 mg/l, PCT ≤ 0,5 ng/ml<br>– No clinical sign of infections<br>– Provision of written informed consent or through a surrogate as appropriate	– Stroke admission > 24 hours after onset of infarct signs<br>– NIHSS < 8<br>– Age < 18 years<br>– Immune suppressive drugs<br>– CRP < 50 mg/l, PCT < 0,5 ng/ml<br>– Clinical sign of infections or clinically significant anaemia<br>– Non-curatively treated malignoma<br>– Severe cerebral disorder in medical history (e.g. ischemic or hemorrhagic stroke, meningitis, encephalitis, severe cerebral trauma or epilepsy<br>– Contraindication for MRI (e.g. pace maker)<br>– Rejection of written informed consent

Key inclusion criteria and follow up of patients are illustrated.

Supplemental Figure I

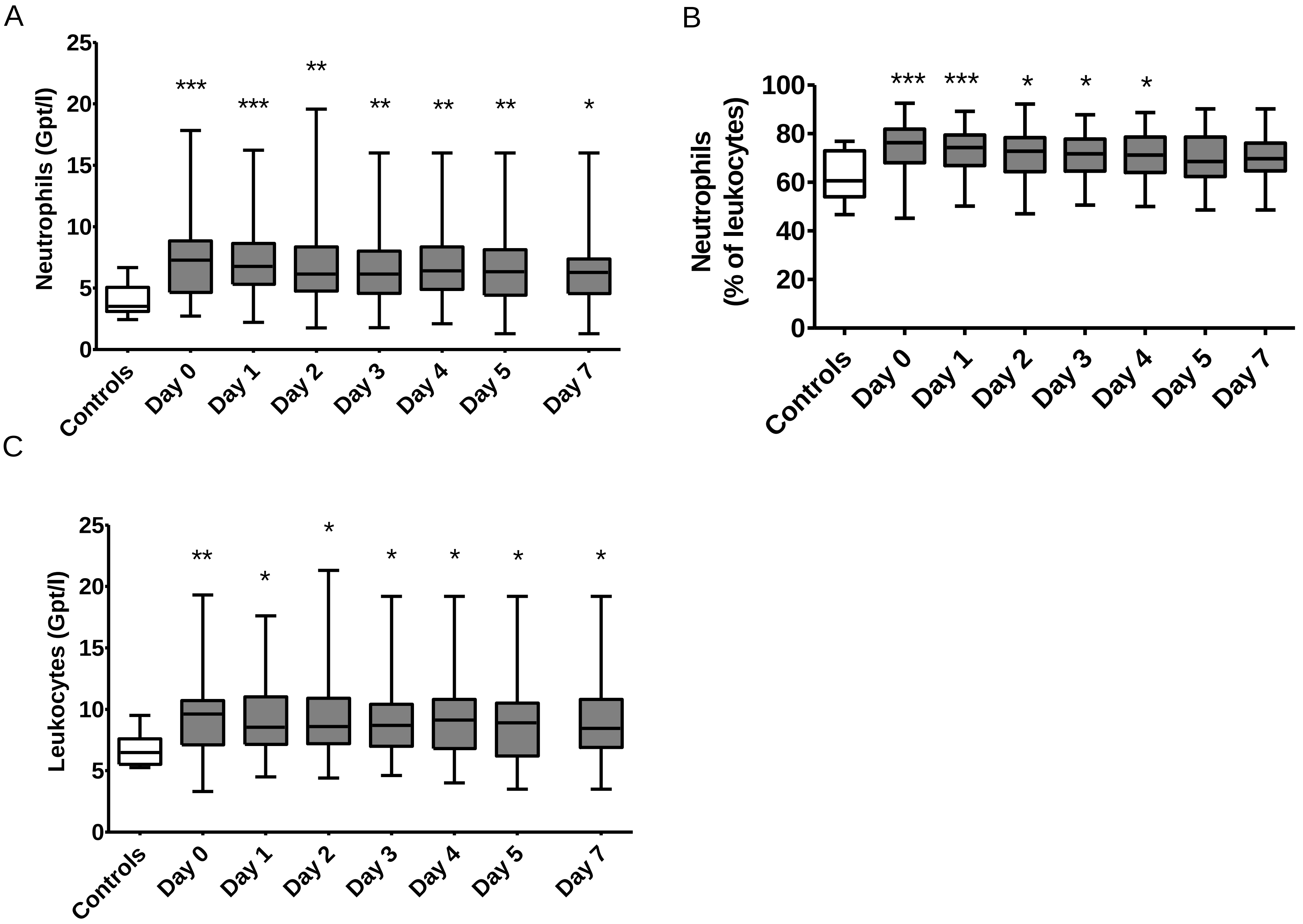

Supplemental Figure II

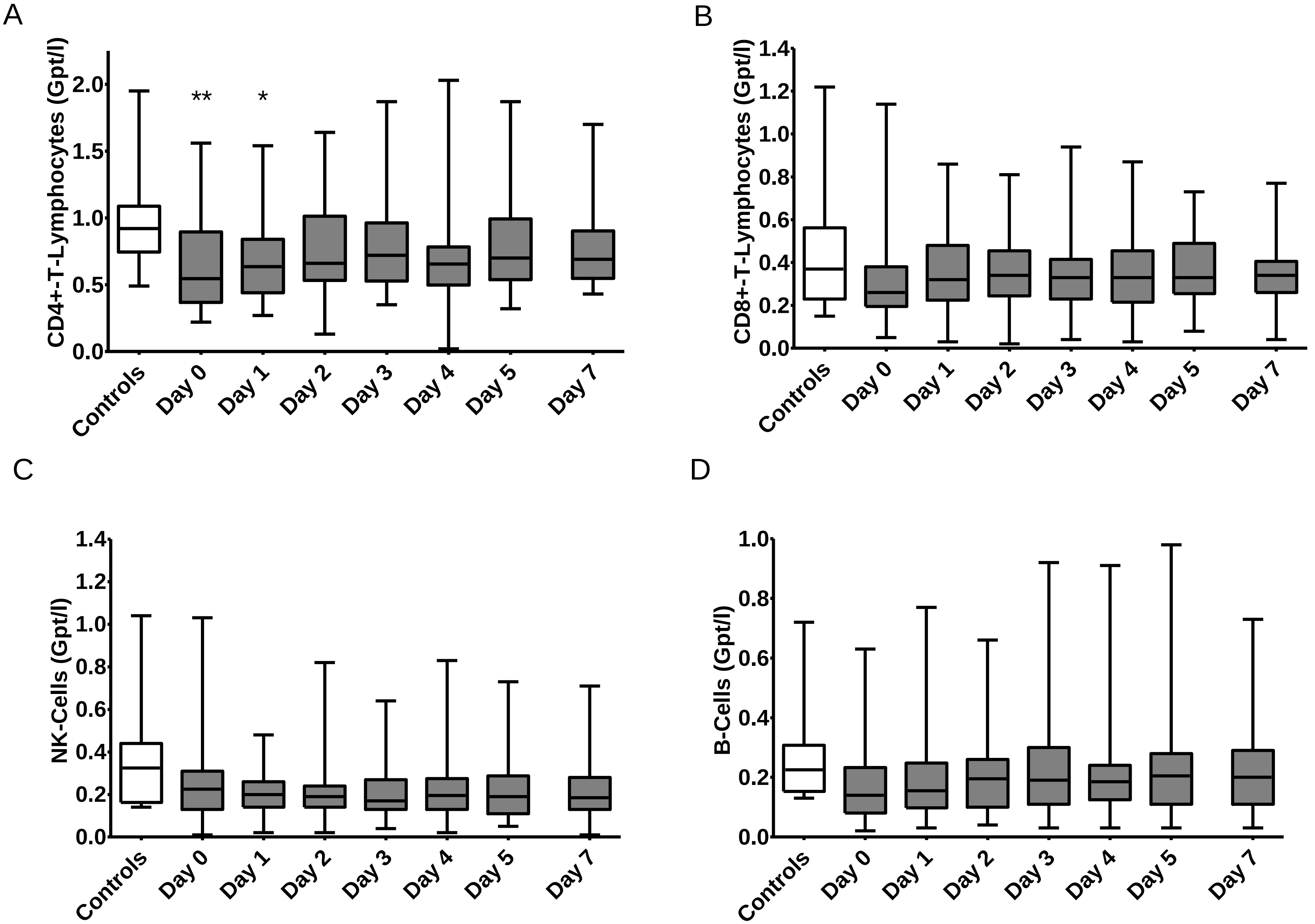

Supplemental Figure III

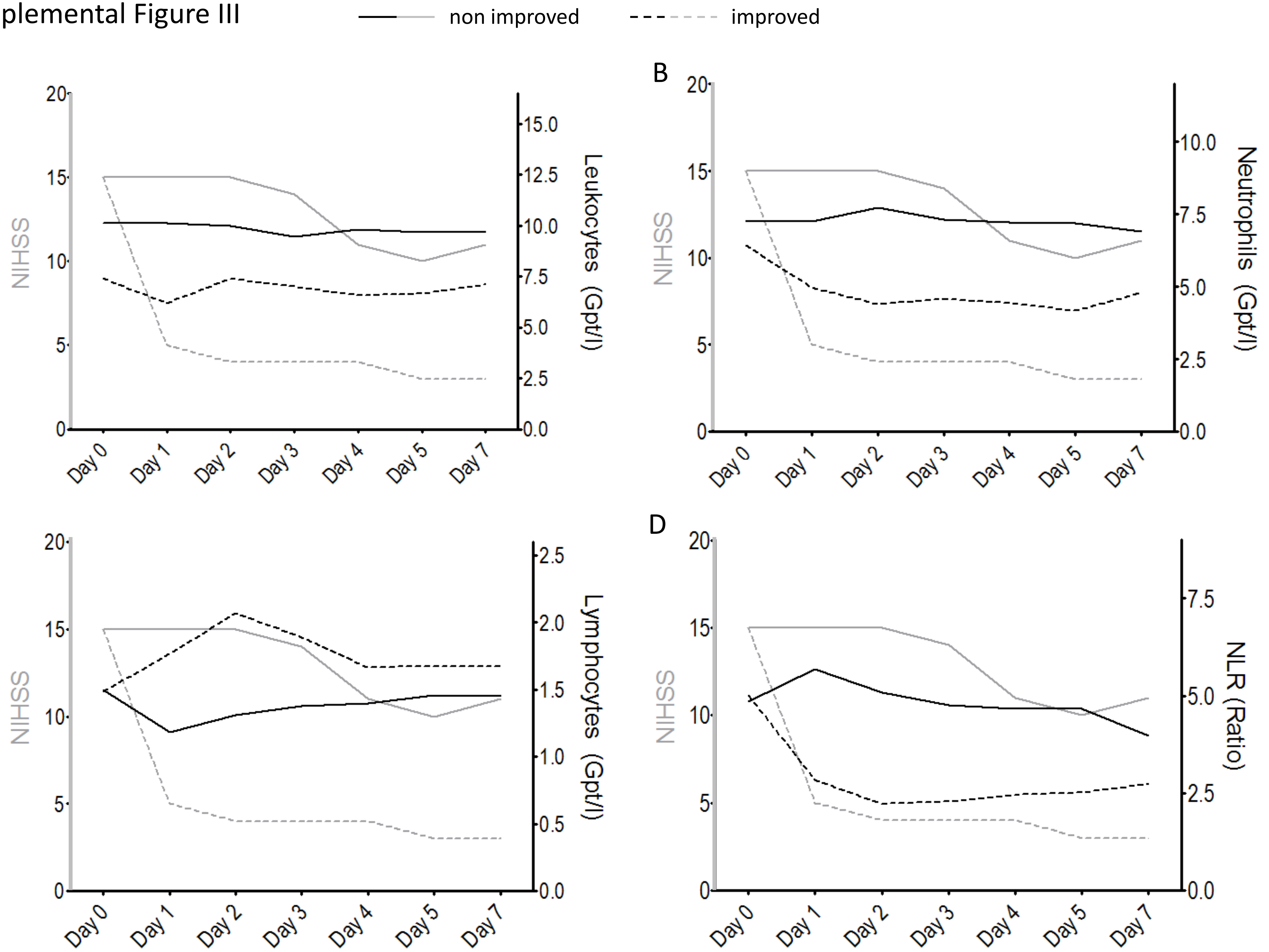

Supplemental Figure IV

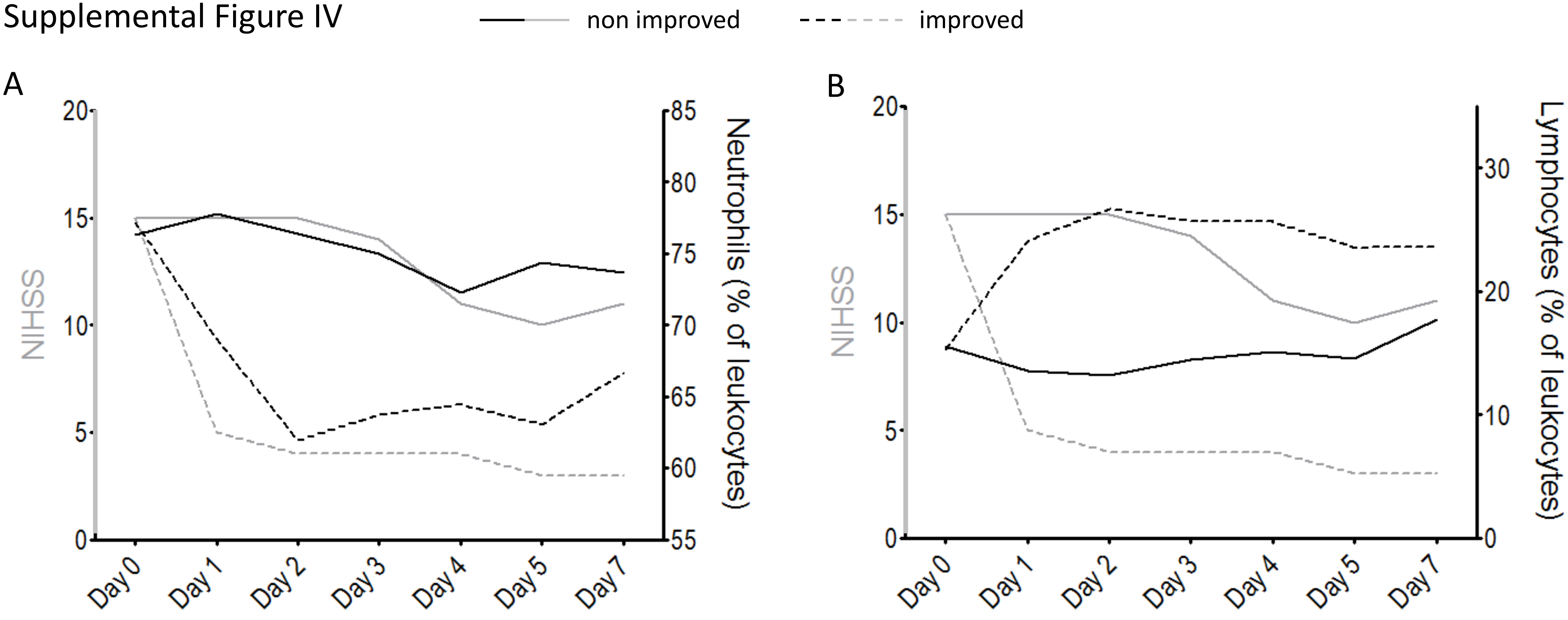

Supplemental Figure Legends

Supplemental Figure I

Comparison of control patients (white bars) and stroke patients (grey bars) on the day of stroke unit admission (day 0), day 1, 2, 3, 4, 5 and day 7 is demonstrated. In (A) absolute neutrophils (Gpt/l), in (B) relative neutrophils (%) and in (C) absolute leukocyte count (Gpt/l) are shown. * $p<0.05$; ** $p<0.01$; *** $p<0.001$; Box and whiskers (Min – Max) are given.

Supplemental Figure II

Comparison of control patients (white bars) and stroke patients (grey bars) on the day of stroke unit admission (day 0), day 1, 2, 3, 4, 5 and day 7 is demonstrated. In (A) absolute numbers of T helper cells (Gpt/l), in (B) absolute number of cytotoxic T cells, in (C) absolute NK cells (Gpt/l) and in (D) absolute numbers of B cells are shown (Gpt/l). * $p<0.05$; ** $p<0.01$. Box and whiskers (Min – Max) are given.

Supplemental Figure III

Progression of median NIHSS-Scores (grey) compared with absolute leukocyte (A, black), neutrophil (B, black), lymphocyte values (C, black) and the neutrophil-lymphocyte ratio (D, black) are shown. Dotted lines represent the patients with improvement, and solid lines represent patients without improvement. Median is given.

Supplemental Figure IV

Progression of median NIHSS-Scores (grey) compared with relative neutrophil (A, blacks) and relative lymphocyte values (B, black) are shown. Dotted lines represent the patients with improvement, and solid lines represent patients without improvement. Median is given.
